# Modified Periodic Acid‐Schiff (PAS) Is an Alternative to Safranin O for Discriminating Bone–Cartilage Interfaces

**DOI:** 10.1002/jbm4.10742

**Published:** 2023-04-21

**Authors:** Kelsey M Kjosness, Philip L Reno, Maria A Serrat

**Affiliations:** ^1^ Department of Bio‐Medical Sciences Philadelphia College of Osteopathic Medicine Philadelphia PA USA; ^2^ Department of Biomedical Sciences, Joan C. Edwards School of Medicine Marshall University Huntington WV USA

**Keywords:** BONE AND CARTILAGE, GROWTH PLATE, HISTOLOGY, HISTOMORPHOMETRY, STAINING METHODS

## Abstract

Cartilage histomorphometry is often performed on decalcified, paraffin‐embedded bone sections, which provide versatility in staining applications from basic morphology to immunohistochemistry. Safranin O is a cationic dye that binds to proteoglycans in cartilage and is routinely used to assess growth plate dynamics and/or fracture repair at bone–cartilage interfaces. When used with a counterstain such as fast green, safranin O can offer exquisite differentiation of cartilage from surrounding bone. However, various decalcification and processing methods can deplete proteoglycans, rendering inconsistent, weak, or absent safranin O staining with indiscriminate bone–cartilage boundaries. We sought to develop an alternative staining methodology that preserves the contrast of bone and cartilage in cases of proteoglycan depletion that can be applied when other cartilage stains are unsuccessful. Here, we describe and validate a modified periodic acid‐Schiff (PAS) protocol that we developed using Weigert's iron hematoxylin and light green stains as an alternative to safranin O for discriminating bone–cartilage interfaces of skeletal tissues. This method provides a practical solution for differentiating bone and cartilage when safranin O staining is not detected after decalcification and paraffin processing. The modified PAS protocol can be useful for studies in which identification of the bone–cartilage interface is essential but may not be preserved with standard staining approaches. © 2023 The Authors. *JBMR Plus* published by Wiley Periodicals LLC on behalf of American Society for Bone and Mineral Research.

## Graphical abstract

Safranin O is a commonly used dye that binds to proteoglycans in cartilage and is routinely used to assess growth plate dynamics and/or fracture repair at bone–cartilage interfaces. Decalcification and processing methods can deplete proteoglycans, rendering weak or absent safranin O staining with indiscriminate cartilage boundaries. This problem‐solving article describes and validates a modified periodic acid‐Schiff (PAS) protocol using Weigert's iron hematoxylin and light green stains as an alternative to safranin O for discriminating bone–cartilage interfaces when other stains fail. This approach may be particularly useful for studies in which samples are difficult to acquire or even irreplaceable.

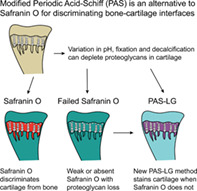

## Introduction

**Fig. 1:**
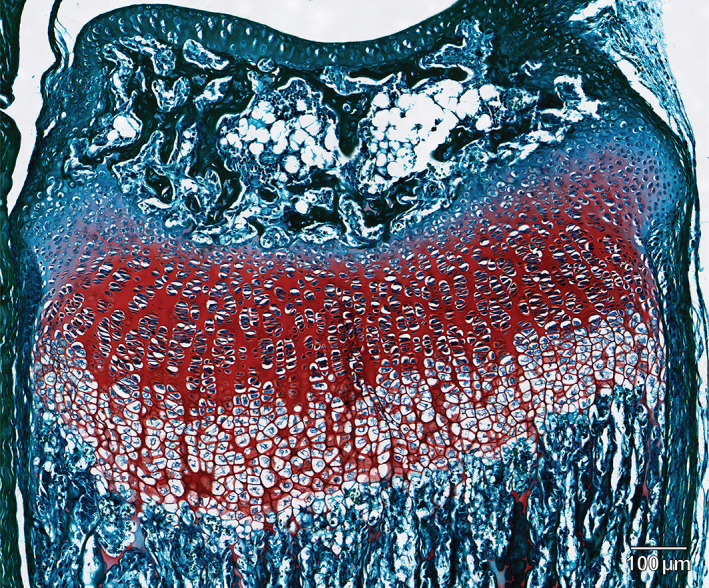
Successful safranin O staining in a 17‐day‐old mouse distal radial growth plate illustrating distinction of cartilage (red) from surrounding bone and soft tissue (green). Scale bar = 100 μm.

Safranin O is frequently used with fast green counterstain to visualize cartilage proteoglycans^(^ ^1^, ^2^ ^)^ because it can produce clearly demarcated bone–cartilage interfaces (Fig. 1).^(^ ^3^ ^)^ Safranin O is a cationic dye that binds stoichiometrically to the anionic glycosaminoglycan component of proteoglycans in solution.^(^ ^4^ ^)^ However, this stoichiometric relationship (color intensity of stain proportional to proteoglycan concentration) is not always maintained in histological sections because of glycosaminoglycan loss.^(^ ^5^, ^6^ ^)^ Weak or absent safranin O staining can result from glycosaminoglycan depletion, even when proteoglycans are still detectable in the cartilage matrix with antibodies, suggesting that safranin O may not be a sensitive indicator of proteoglycan content at low levels.^(^ ^6^, ^7^ ^)^

Proteoglycans and/or their glycosaminoglycan component can be lost from cartilaginous tissues in multiple ways. Proteoglycans are soluble and prone to leaching during submersion in fixatives, buffers, and decalcifying solutions. Many studies have reported strong safranin O staining regardless of fixation, decalcification, or sectioning procedures.^(^ ^8^, ^9^ ^)^ However, others have experienced weak or absent safranin O staining, presumably attributed to depletion of proteoglycans and/or their glycosaminoglycan component during tissue processing.^(^ ^10^, ^11^, ^12^, ^13^ ^)^ For example, glycosaminoglycan loss was evident after fixation in 4% formaldehyde and decalcification with 10% EDTA, with most of the loss having occurred during the decalcification step.^(^ ^12^ ^)^ Other studies have shown that safranin O staining intensity varies with decalcification solution and/or temperature,^(^ ^14^, ^15^ ^)^ as well as fixation time,^(^ ^13^ ^)^ suggesting that glycosaminoglycans can progressively leach from samples. Even short exposure to isotonic buffers can extract proteoglycans from tissues. In fact, most of this leaching, ranging from 10% to 30% of total proteoglycan content, can occur within the first 30 minutes of rinsing time.^(^ ^16^, ^17^ ^)^

While performing routine processing and staining of juvenile mouse bones, we detected inconsistencies in safranin O intensity in some of our specimens. Although safranin O staining typically renders a clear demarcation of the cartilage–bone interface when counterstained with fast green (Fig. 1), we found that some specimens exhibited unexpectedly weak or absent safranin O stain and it was not always possible to predict when this failure of safranin O stain would occur. These findings were problematic for morphometric analyses of the growth plate because we relied on the metachromatic color of safranin O to consistently discriminate the cartilage boundary, and many of our samples were irreplaceable. Although we followed the same protocols, our literature review revealed that minor differences in pH between batches of reagents for fixation, decalcification, and washes, as well as differences in submersion time to accommodate different‐sized samples and/or time between fixation and processing, could underlie the weak or absent safranin O staining that we observed. For example, there was a delay in processing some of our samples because of equipment failure and several batches of 4‐ to 6‐week‐old decalcified mouse knees were kept in 70% ethanol for approximately 6 weeks. When the samples were processed, most exhibited weak to absent safranin O staining despite otherwise following all the same protocols.

Metachromatic staining methods, such as safranin O, create a color change in certain tissues because the dye forms high‐affinity salt linkages with polyanions, such as the chondroitin sulfate groups in cartilage.^(^ ^6^ ^)^ For example, toluidine blue also has a high affinity for sulfate groups in proteoglycans and turns cartilage a deep purplish‐blue color in contrast to the light blue of surrounding noncartilaginous tissues.^(^ ^18^ ^)^ Such metachromatic approaches may therefore be unreliable when polyanions leach out during fixation and processing protocols.^(^ ^18^ ^)^ Results of diminished safranin O staining are seldom published when not a direct result of decreased proteoglycan content due to genetic mutation or disease state. We are aware, however, of other colleagues who have experienced this problem through conversation and requests for help troubleshooting when standard safranin O methods failed to stain their tissues as expected. To address this problem, we sought to develop an alternative staining methodology that preserves the metachromatic contrast of bone and cartilage even in cases of proteoglycan depletion.

Periodic acid‐Schiff (PAS) is a carbohydrate‐specific staining reaction used to demonstrate glycogen and other polysaccharides,^(^ ^6^, ^19^ ^)^ such as the mucopolysaccharides in cartilage.^(^ ^2^ ^)^ This two‐step protocol involves exposure to periodic acid, which oxidizes glycol groups to aldehydes, followed by Schiff's reagent, which turns the aldehydes a rose‐violet color.^(^ ^2^, ^6^, ^19^ ^)^ Although the glycosaminoglycan component of proteoglycans does react with PAS after prolonged incubation, PAS preferentially targets glycol groups with a neutral charge, making this reaction more sensitive than safranin O for staining cartilage matrix.^(^ ^8^, ^20^ ^)^ Here we describe a simple and novel modification of a standard PAS protocol using Weigert's iron hematoxylin and light green stains that produces a contrast between bone (blue‐green) and cartilage (purple). This technique provides a clear distinction of the bone–cartilage interface even when safranin O staining does not.

## Materials and Methods

All animal procedures were approved by and followed all ethical guidelines of the Institutional Animal Care and Use Committees at Northeast Ohio Medical University (formerly Northeastern Ohio Universities College of Medicine, protocol 05‐008) and Pennsylvania State University (protocol 44735‐1). A total of 87 juvenile mice were used in this study. Knees were harvested from 37 male CF‐1 outbred mice (Charles River Laboratories, Wilmington, MA, USA) between 4 and 6 weeks old as part of another investigation,^(^ ^21^ ^)^ and their tibial growth plate sections were used to develop the PAS protocol described here. In a separate laboratory, forelimbs were harvested from 50 C57BL/6 and FVB/NJ juvenile mice (The Jackson Laboratory, Bar Harbor, ME, USA) between 9 and 40 days old as part of other investigations. Their distal radial growth plate sections were used for subsequent protocol validation.

Bones for protocol development were fixed in 10% neutral buffered formalin for 24 hours and decalcified in 10% EDTA for 4 weeks, rinsed, and processed following our published methods.^(^ ^22^ ^)^ Because of unexpected equipment failure, decalcified and rinsed samples were placed in 70% ethanol for approximately 6 weeks before processing. Bones used in protocol validation were fixed in 4% paraformaldehyde in 1× phosphate buffered saline (PBS) for 24 to 48 hours and decalcified in 10% EDTA for 1 to 4 weeks (based on tissue size and specimen age).^(^ ^23^ ^)^ Samples for both protocol development and validation were then rinsed in PBS, dehydrated in a graded series of ethanol, cleared in Histoclear II (National Diagnostics, Atlanta, GA, USA), CitriSolv (Thermo Fisher Scientific, Waltham, MA, USA), or xylene, embedded in Paraplast Plus paraffin, and cut into 6 to 8 μm sections using a rotary microtome. Because of weak or absent safranin O staining in growth plate cartilage, sections were stained using a modified PAS protocol that we developed to provide sufficient contrast for visualizing the cartilage–bone interface as detailed below. Additional sections were stained using a standard PAS protocol following manufacturer instructions for a commercially available kit (Sigma‐Aldrich, St. Louis, MO, USA; Periodic Acid‐Schiff Kit, cat. no. 395B) and with 0.1% thionin to visualize cartilage histology.^(^ ^19^ ^)^ Safranin O staining followed previously published methods.^(^ ^23^ ^)^

Modified PAS staining was performed at room temperature using a commercially available kit (Sigma‐Aldrich, Periodic Acid‐Schiff Kit, cat. no. 395B) with several modifications to the manufacturer's protocol. For this protocol, the key reagents in the staining kit are periodic acid (1 g/dL; Sigma‐Aldrich, cat. no. 3951‐100 ML) and Schiff's reagent (pararosaniline HCl 1% and sodium metabisulfite 4% in hydrochloric acid 0.25 mol/L; Sigma‐Aldrich, cat. no. 3952‐50 ML). Care must be exercised when applying this method as periodic acid is corrosive, Schiff's reagent is toxic, and hematoxylin is harmful (flammable liquid and skin irritant), according to the manufacturer SDS information. Solutions should also be prepared fresh and/or used within manufacturer expiration dates for optimal results.

The first part of the procedure closely followed the manufacturer's instructions: Deparaffinized and hydrated sections were exposed to periodic acid for 5 minutes, rinsed in three changes of distilled water, immersed in Schiff's reagent for 10 minutes (time was reduced from the manufacturer protocol of 15 minutes to enable a better counterstain), and then rinsed under running distilled water for 5 minutes. The remaining steps were novel to this study: Sections were then counterstained with Weigert's iron hematoxylin (detailed in Presnell and Schreibman^(^ ^19^ ^)^) for 5 minutes, rinsed in two changes of distilled water (2 minutes each), “blued” in 1× PBS for 20 seconds, briefly rinsed in distilled water, and stained with a 1% light green solution (Sigma‐Aldrich, cat. no. L1886) for 30 seconds to 2 minutes, depending on the preferred intensity of the stain. Slides were then rinsed in distilled water for 2 minutes, allowed to briefly air dry, then finally dehydrated, cleared, and cover‐slipped.

Slides for protocol development were imaged using a Leica (Buffalo Grove, IL, USA) DM2500 microscope coupled with a QImaging Retiga R6 6.0 megapixel color camera (Surrey, BC, Canada) interfaced to a PC running Ocular Software (version 2.0). Sections used for protocol validation were imaged with a Motic (San Francisco, CA, USA) Easy Scan digital slide scanner and Motic VM 3.0 Motic Digital Slide Assistant for image capture.

## Results

**Fig. 2:**
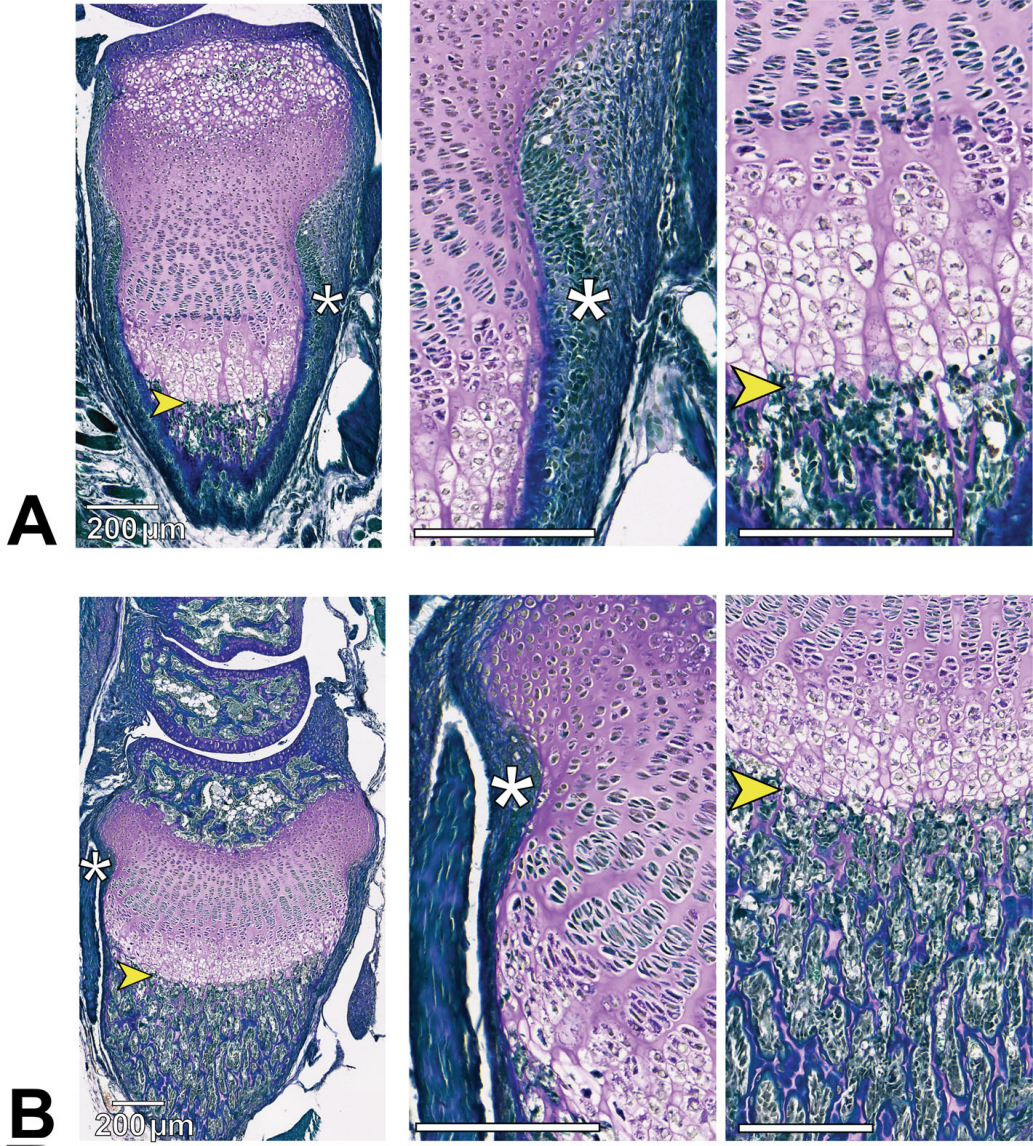
Modified periodic acid‐Schiff (PAS) staining of distal radial growth plates from (*A*) 9‐day‐old and (*B*) 15‐day‐old mice stained as part of our protocol validation. Articular cartilage of the radiocarpal joint (distal) is at the top in all images. Magnified panels show demarcation of cartilage at the perichondrium (asterisks) and metaphyseal chondro‐osseous junction (yellow arrowheads). Calcified cartilage at the metaphyseal chondro‐osseous junction is also stained purple. The secondary center of ossification is present in the 15‐day‐old mouse in (*B*), further delineating the growth plate between epiphyseal (top) and metaphyseal (bottom) bone. Scale bar = 200 μm for all images.

Our modified PAS protocol using Weigert's iron hematoxylin and light green stains (PAS‐LG) produced a distinctive purple staining in cartilage, which was clearly demarcated from surrounding bone and soft tissue that appeared a blue‐green color (Fig. 2). Images shown in Fig. 2 are distal radial growth plates from 9‐day‐old (Fig. 2*A*) and 15‐day‐old (Fig. 2*B*) mice stained with PAS‐LG as part of our protocol validation. Magnified panels in Fig. 2 show that cartilage is clearly demarcated from perichondrium (asterisks in Fig. 2) as well as the metaphyseal chondro‐osseous junction (yellow arrowheads in Fig. 2), validating the utility of this method for discriminating cartilage from bone and other surrounding tissue. Calcified cartilage at the metaphyseal chondro‐osseous junction is also stained purple (Fig. 2).

**Fig. 3:**
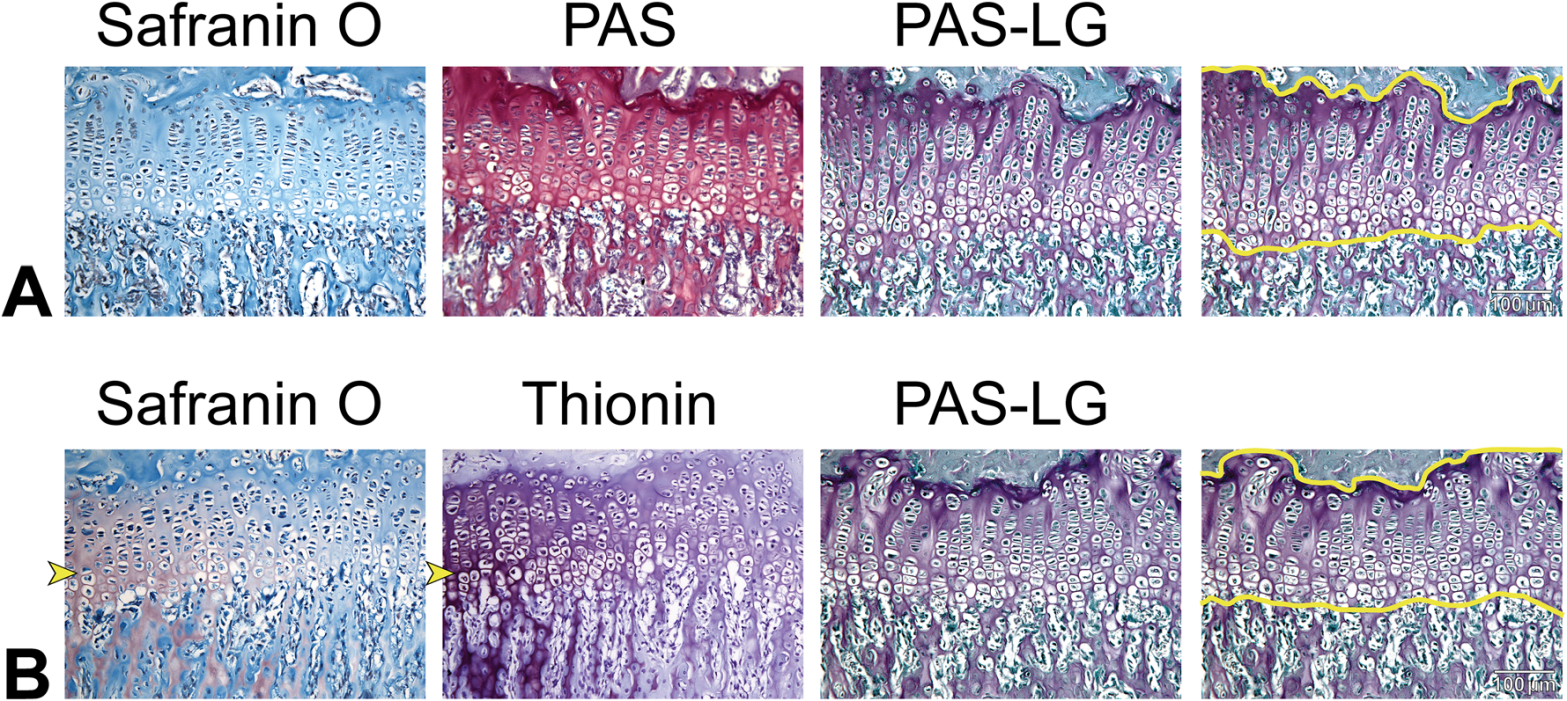
Comparison of the modified periodic acid‐Schiff (PAS) method with other cartilage stains on serial sections of proximal tibial growth plates from two different 6‐week‐old mice. (*A*) Safranin O (left) did not stain the growth plate, making the bone cartilage interface indistinct. Standard PAS staining (middle) did stain cartilage, but the color was difficult to discriminate from the surrounding bone because all tissues stained reddish‐purple. The modified PAS protocol using Weigert's iron hematoxylin and light green (PAS‐LG, right) stained cartilage purple and surrounding bone and soft tissue a blue‐green color. This enabled multiple users to consistently trace the boundaries of the cartilage (far right) in a way that was not possible in the failed safranin O image. (*B*) In a different mouse, the tibial section was weakly stained with safranin O toward the lateral edge of the growth plate as indicated by the yellow arrowhead (left), whereas safranin O was absent toward the inner edge of the growth plate. Serial sections stained with thionin (middle) match this pattern of metachromasia, suggesting an uneven loss of proteoglycans across the tissue (more proteoglycans retained toward the lateral edge marked by the arrowheads). The PAS‐LG section shows a clear bone‐cartilage distinction across the entire image (right) and reliable demarcation of the growth plate boundary for data collection (far right). Scale bar = 100 μm for all images.

Most importantly, we were able to discriminate bone–cartilage interfaces using modified PAS staining on sections that were not successful with other cartilage stains. We found during protocol development that our modified PAS‐LG protocol renders a clear bone–cartilage distinction and robust staining in specimens for which safranin O failed and standard PAS staining produced limited distinguishing features between cartilage and bone (Fig. 3). Under standard protocols, a positive PAS reaction produces a rose to purplish‐red color.^(^ ^19^ ^)^ When applied to our bone sections, we found that growth plate cartilage appeared a deep red‐purple color and surrounding bone was pale red‐purple with a somewhat indiscriminate boundary (Fig. 3*A*, middle).

**Fig. 4:**
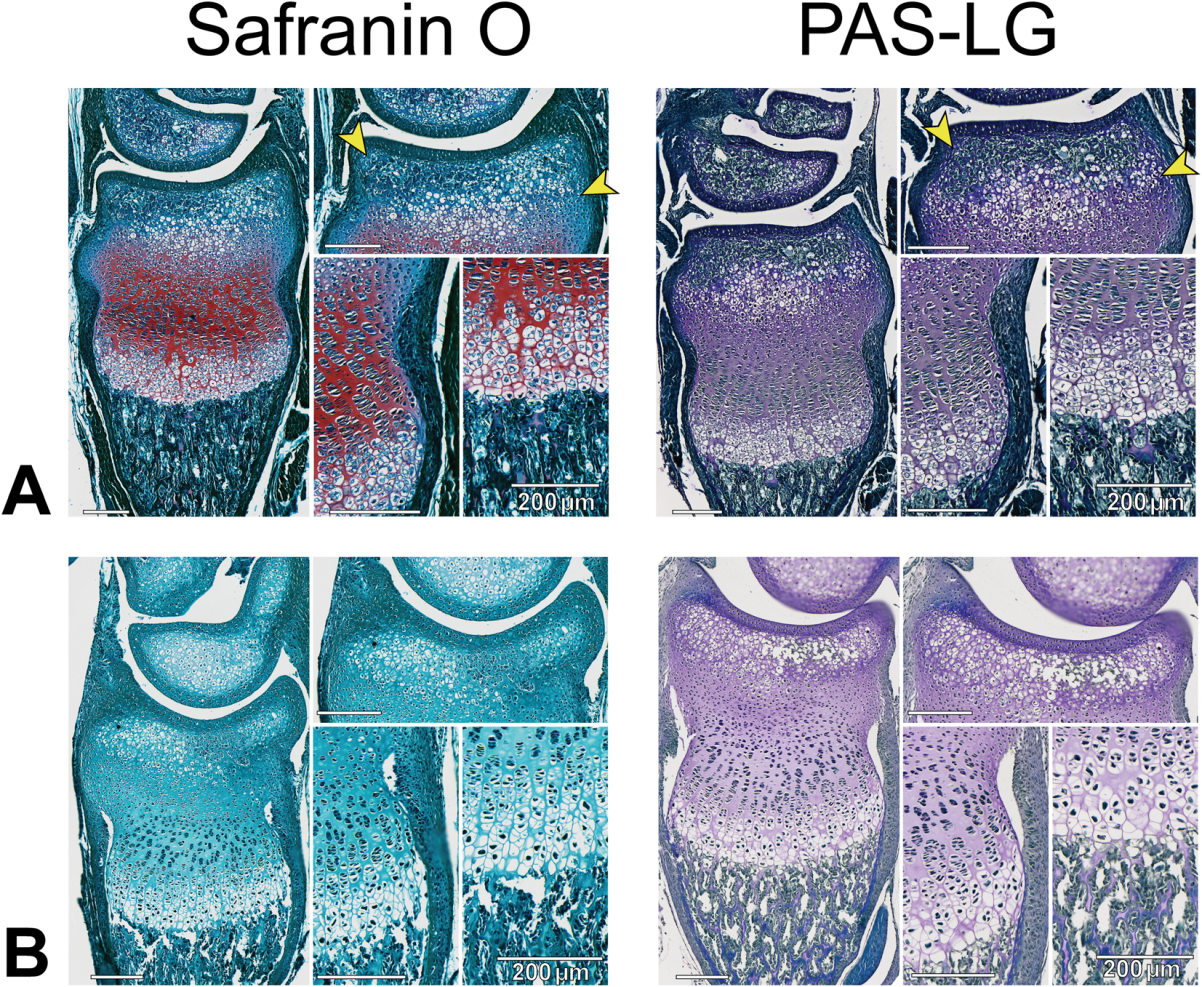
Comparison of safranin O and periodic acid‐Schiff protocol using Weigert's iron hematoxylin and light green (PAS‐LG) staining in distal radial growth plates of two different 9‐day‐old mice. Orientation and morphology match that described in Fig. 1. (*A*) Although safranin O did render the expected red staining of cartilage, the stain did not extend to boundaries of the perichondrium or epiphyseal cartilage nearest the articular surface (yellow arrowheads). The same cartilaginous areas did stain purple using the modified PAS protocol as shown by the yellow arrowheads on the right. A different mouse (*B*) did not stain red with safranin O, but all cartilage did stain purple with a blue‐green bone interface using the modified PAS protocol. Magnified panels demonstrate articular cartilage, growth plate, perichondrium, and developing secondary center of ossification as in Fig. 1. Scale bar = 200 μm in all images.

Figure 3 shows the application of our PAS‐LG method on serial sections of proximal tibial growth plates from two different 6‐week‐old mice with weak and/or absent safranin O staining. In Fig. 3*A*, safranin O (left) did not stain the growth plate at all, making it nearly impossible to differentiate cartilage from bone. However, standard PAS staining (middle) showed a clear staining pattern in cartilage, but the color distinction from bone was vague because all parts of the tissue had a purple‐red hue. When we applied our modified PAS protocol using Weigert's iron hematoxylin and light green, cartilage in the PAS‐LG image (right) appeared purple, whereas the surrounding bone and soft tissue was blue‐green. This allowed independent investigators to outline the same boundaries of the growth plate (Fig. 3*A*, far right) for consistent data collection, which we were unable to do reliably in the failed safranin O sections because of the vague cartilage–bone interface. In Fig. 3*B*, the tibial section was weakly stained with safranin O toward the lateral edge of the growth plate (shown by the yellow arrowhead on the left), while safranin O was absent toward the inner edge of the growth plate. Serial sections stained with thionin (middle) match this pattern of metachromasia at the same lateral edge (shown by yellow arrowhead) with no color distinction in cartilage toward the inner edge of the growth plate, suggesting an uneven loss of proteoglycans across the tissue (more proteoglycans retained toward the lateral edge marked by the arrowheads). However, the serial‐stained PAS‐LG section in Fig. 3*B* shows a clear bone–cartilage distinction across the entire image, enabling consistent tracing of the growth plate boundary for data collection (far right). Proximal tibial growth plates of all 37 mice used in our protocol development successfully stained with PAS‐LG in a similar pattern, despite weak or absent safranin O staining in all individuals. Furthermore, not only did all 50 sections used in protocol validation stain with PAS‐LG regardless of safranin O success, we found that our modified PAS‐LG protocol actually provided a better distinction of the bone–cartilage interface in some cases (Fig. 4).

In specimens where safranin O staining was successful and produced the expected red staining of cartilage (Fig. 4*A*), this staining did not always extend to boundaries of the perichondrium or epiphyseal cartilage nearest the articular surface (yellow arrowheads in Fig. 4*A*). However, these cartilaginous areas did stain purple using the modified PAS protocol (yellow arrowheads in Fig. 4*A*). In specimens for which no red staining occurred with safranin O (Fig. 4*B*), all cartilage did stain purple using the modified PAS protocol (Figs. 3 and 4*B*). We could not always predict when safranin O would fail in our samples, yet we had a 100% success rate using the PAS‐LG protocol.

## Discussion

This protocol was established as an operable alternative to the safranin O method for differentiating bone and cartilage, wherein cartilage appears purple and the surrounding bone and soft tissue stains blue‐green. We were able to capitalize on the sensitivity of the PAS reaction for staining cartilage with low proteoglycan content, while incorporating a novel combination of Weigert's iron hematoxylin and light green counterstains to provide a distinctive bone contrast.

This modified PAS protocol provides a suitable and consistent method for assessing cartilage morphology independent of proteoglycan content, and importantly, under different fixation (neutral buffered formalin and paraformaldehyde) and processing/clearing (CitriSolv, Histoclear II, xylene) conditions that we tested. It produces reliable visualization of the bone–cartilage interfaces at the metaphyseal chondro‐osseous junction, subchondral bone interface, and perichondrial bone collar. We believe this method is successful because PAS targets glycol groups that are not impacted by tissue fixation and processing protocols.

Other studies have reported novel combinations of staining methods to distinguish bone from cartilage, including the bone‐inflammation‐cartilage (BIC) stain, which is intended to distinguish cartilage degradation and inflammation from bone erosion^(^ ^24^ ^)^; however, the BIC protocol utilizes safranin O to stain cartilage and is therefore sensitive to proteoglycan depletion. We specifically designed the modified PAS protocol here to stain cartilage for morphological studies, regardless of proteoglycan content.

While the absence of safranin O staining is typically only reported when it is an indicator of proteoglycan loss due to disease or genetic mutation, it is a surprisingly common and underrecognized artifact of many tissue processing protocols. This can be particularly problematic when samples span a range of ages and sizes in developmental studies that would otherwise require time‐consuming optimization to avoid potential proteoglycan leaching between ages during decalcification. In addition to the data presented here for development and validation of this modified PAS protocol, we have shared our methods with several other colleagues who have successfully stained their specimens using the modified PAS technique when safranin O did not stain cartilage. Although there are commercially available kits using light green with PAS, such as Abcam (Cambridge, MA, USA) Periodic Acid‐Schiff (PAS) Stain Kit (Mucin Stain) (ab150680), our protocol is distinct in the application of Weigert's iron hematoxylin and light green counterstains to specifically highlight the bone–cartilage interface.

In summary, this modified PAS protocol provides a novel method for staining cartilage when other cartilage‐specific stains are unsuccessful. Although not intended to replace classic methods such as safranin O, our protocol is an alternative for discriminating bone–cartilage interfaces when other approaches fail. The PAS method consistently renders purple staining in cartilage and blue‐green staining in bone, independent of proteoglycans, which are the target of most other cartilage stains. The protocol could have important uses for studies in which identification of the bone–cartilage interface is essential, but may not be preserved because of proteoglycan depletion, whether from tissue processing or genetic disease. This approach offers an especially useful alternative for staining cartilage when standard methods fail in irreplaceable samples.

## Author Contributions

**Kelsey M Kjosness:** Data curation; formal analysis; investigation; methodology; validation; visualization; writing – original draft; writing ‐ review and editing. **Philip L Reno:** Data curation; formal analysis; funding acquisition; methodology; supervision; validation; visualization; writing – review and editing. **Maria A Serrat:** Conceptualization; data curation; formal analysis; funding acquisition; investigation; methodology; project administration; resources; supervision; validation; visualization; writing – original draft; writing – review and editing.

## Conflicts of Interest

The authors declare that they have no conflicts of interest.

### Peer Review

The peer review history for this article is available at https://www.webofscience.com/api/gateway/wos/peer-review/10.1002/jbm4.10742.
